# HIV, visceral leishmaniasis and Parkinsonism combined with diabetes mellitus and hyperuricaemia: A case report

**DOI:** 10.1186/1757-1626-1-183

**Published:** 2008-09-25

**Authors:** Krishna Pandey, Prabhat Kumar Sinha, Vidya Rabidas, Nawin Kumar, Sanjiva Bimal, Neena Verma, Chandrasekhar Lal, Pradeep Das

**Affiliations:** 1 Rajendra Memorial Research Institute of Medical Sciences (Indian Council of Medical Research), Agamkuan, Patna, – 800 007, India

## Abstract

**Background:**

Visceral leishmaniasis is caused by a protozoan parasite, *Leishmania donovani *and transmitted by the bite of female sandflies. India is endemic for this disease. On the other hand, India contributes to the largest number of cases of HIV as well.

**Case presentation:**

We hereby report an unusual case presentation of Visceral leishmaniasis/HIV co-infection with additional features of Parkinsonism and hyperuriciemia in an Indian male patient aged about 50 years.

**Conclusion:**

The increasing incidence of HIV/VL co-infection in India is of utmost importance. The diagnostic and management aspects of these cases are very difficult to handle particularly in an underdeveloped country like India.

## Background

Visceral leishmaniasis (VL) is a disease of prime public health importance in a developing country like India. Similarly, Human Immuno-deficiency Virus (HIV) infection is also assuming a dangerous proportion in this country. The disease combination of these two is a deadly one. In Europe it is estimated that approximately 25–70% of adult cases of VL are HIV co infected [1]. Leishmaniasis is the third most frequent opportunistic infection in HIV patients worldwide [2]. The lack of poor reporting system and diagnostic facilities leads to under reporting of these diseases.

It is estimated that 350 million people are at risk of VL with an incidence of 0.5 million and a prevalence of 2.5 million worldwide. About 90% of these cases occur in Indian sub-continent, Brazil and Sudan. 80% of the Indian figures occur in Bihar State alone [3]. Since the advent of HIV/AIDS in 1981 about 25 million people have died of this disease. In 2007 globally about 2 million people died of AIDS, 33 million were living with HIV and about 2.5 million people were newly infected with the virus. Although the proportion of people living with HIV in India is lower than previously estimated, the epidemic continues to affect about 2.5 million people [4]. We present here an extremely interesting case report of a combination of these diseases along with Parkinsonism, Diabetes mellitus and Hyperuricaemia.

## Case presentation

A 50 year old male from a VL endemic village of Bihar presented to the OPD of this institute in August 2007. He had fever with weakness and pallor of about 3 months duration. The fever was associated with chills. He also complained of frequent loose motions in the last 6 months and had lost about 7 kg of weight. He had dry cough of one month duration associated with small amount of haemoptysis and oral ulceration more marked on the tongue. He also complained of frequent micturition and pain in the metatarsophalangeal joints of the big toes bilaterally alongwith some amount of generalized joint pain.

He was a truck driver by profession and was a frequent visitor to the red light areas of metropolitan cities namely Mumbai and Kolkata. On close questioning he revealed having frequent sex with multiple commercial sex workers. He use to take alcohol, toddy and smoked 2–3 cigarettes per day. He had two small children aged 8 years & 6 years (both girls). At the time of presentation he had bradykinesia with mask facies and characteristic pill rolling tremor more marked on the right side. The arms swing was also absent on the right side while walking and his gait was festinant. There were no features of forgetfulness or dementia, history of falls or any associated autonomic disturbances. He however had micrographia with monotonous speech.

On clinical examination he had a mouth temperature of 102°F (39°C) with a pulse rate of 120/min. His respiration rate was 20/min and his blood pressure was 110/70 mmHg in the left upper limb in supine posture. Chest and cardio vascular system examination were normal except for sinus tachycardia. His liver and spleen were palpable 2 cm and 4 cm below the respective coastal margins in the mid axillary lines. On central nervous system examination the mask facies, bradykinesia, pill rolling tremor, cogwheel rigidity and festinant gait were elicited. Bradykinesia and rigidity were asymmetric. The palmo-mental and glabellar tap reflexes were positive bilaterally. The planters were bilaterally flexor. All the above symptoms were more marked on the right side.

The patient was subjected to various hematological and biochemical investigations along with splenic aspiration, chest x-ray postero-anterior view, ultra sonography of abdomen and computerized tomography (CT scan) of the brain. He had a hemoglobin level of 6 gm/dl along with a total count of 4000 cells/mm^3^. Liver and renal function tests were normal. His fasting blood sugar was 120 mg/dl and post prandial level was 160 mg/dl. His serum uric acid was 7.2 mg/dl. Western Blot for HIV1 was positive. His CD4 count was 180/μl and CD8 count was 643/μl. Splenic aspirate for Leishman Donovan bodies was 3+ according to WHO criteria. Ultra sound showed hepatosplenomegaly with features of fatty liver. CT scan of the brain and ECG were normal. ELISA and PCR for tuberculosis were negative. MRI and spinal fluid examination were also done and found normal.

Based of the above findings a diagnosis of HIV1, VL and Parkinsonism was made. Other associations were diabetes mellitus and hyperuricaemia. He was put on diabetic diet and started on Miltefosine 50 mg capsules twice daily for 28 days after meals along with iron and folic acid supplements. He was also administered Allopurinol in the dose of 100 mg tablets twice daily. He was started on highly active antiretroviral therapy with two nucleoside reverse transcriptase inhibitors namely Zidovudine (200 mg) plus Lamivudine (150 mg) and one non nucleoside reverse transcriptase inhibitor namely Nevirapine 200 mg twice daily after food. Parkinson's disease was treated with Entecapone (100 mg), levodopa (100 mg) and Carbidopa (25 mg) combination twice daily with Triphenhexidyl (2 mg) twice daily along with Selegiline hydrochloride 5 mg twice daily. Other D2 receptor agonists like ropinirole or pramipexole were not added.

After one month of treatment his spleen had regressed, there was no fever and no LD bodies were seen in the bone-marrow aspirate. He was continued on antiretroviral therapy (ART) and Anti-Parkinsonian therapy (entekapone, levodopa, carbidopa, triphenhexidyl and selegeline) along with allopurinol. His CD4 increased to 300/ml. He was able to walk with considerably less tremor. He, however, relapsed for visceral leishmaniasis after 3 months of therapy and was treated with Amphotericin B in the dose of 1 mg/kg body weight for 15 days in 5% dextrose intravenous infusion on alternate days. He was told to report after one month but was ultimately lost to follow up. About 6 months later, it was gathered from his relatives that he had died. VL, itself, is a disease of poorest of the poor as it mainly affects the low socio-economic group and the combination of HIV and Parkinsonism makes it more difficult for the people of poor countries likes India for proper treatment compliance and regular follow-up visits. It is hoped that possibly due to this reason the patient could not turn up and eventually he might have contracted some other AIDS related complications and lost his life. We really feel pity for his poor family.

## Discussion

The treatment and diagnosis of the combination of diseases mentioned above is a very difficult one. As regards the diagnosis of VL, the demonstration of LD bodies in the splenic aspirates along with strip test like rK39 and the relatively new nested PCR can be of great help. Treatment is very difficult in a setting of HIV combination, as no drug along with dosage has been authenticated, there are frequent relapses and drug interactions pose a very difficult challenge [5].

Sodium Antimony Gluconate (SAG) is developing resistance and unresponsiveness to the said drug has been reported to be about 43% from Bihar [6]. Pentamidine particularly because of its side effects has been discarded. Amphotericin B is a much better option but requires hospitalization and monitoring of serum electrolytes mainly potassium. Miltefosine or Hexadexylphosphocholine has completed Phase III and Phase IV and is considered to be a good drug with an initial cure rate of about 94% [7,8]. However it cannot be given in pregnant women being an antineoplastic agent. Sitamaquine, a primaquine analogue is in Phase II trial stage. Another drug an aminoglycoside namely Paromomycin has completed Phase III trial and has a cure rate of about 95% [9]. Phase IV trials of the drug are under way.

Various researchers have tried a combination of these drugs in HIV/VL co-infected cases. This seems to be a good option as there are frequent relapses However; these combinations can lead to an increase in the toxicity [10]. Combinations like Paromomycin with Miltefosine, Amphotericin B with Miltefosine, SAG with Miltefosine, SAG with Paromomycin, Liposomal Amphotericin B with Miltefosine, Amphotericin B with Paromomycin etc are being tried. ART has to be given along with treatment of VL. Nevirapine is not to be given with antituberculous drugs like Rifampicin. Protease inhibitors have various drug interactions and are very costly. Recently fusion inhibitors are coming up in the world market but their main limitation is extremely high cost. Another group of drugs, the integrase inhibitors are also being tried.

Movement disorders are potential neurological complications of AIDS. Dopaminergic dysfunction and the predilection of HIV infection to affect subcortical structures are thought to underlie the development of movement disorders [11]. Extrapyramidal symptoms similar to Parkinson's disease have been reported with retonavir/indinavir and risperidone which have to be used in HIV patients with psychosis [12]. The treatment of Parkinsonism requires Levodopa and Carbidopa combination along with mono amine oxidase B inhibitors like Selegiline and anti-cholinergics like Triphenhexydil. Newer drugs like Entecapone (catechol-o-methyl transferase inhibitors) extend the half life (plasma) and the duration of L-dopa effect by preventing its breakdown. Dopamine (D2) receptor agonists like Ropinirole and Pramipexole are also being tried but are costly and have multiple side effects like excessive sleepiness, dyskinesia and hypotension etc.

## Conclusion

This case report presents new interesting features. Firstly the combination of HIV and VL is increasing and it is very difficult to diagnose and treat. Secondly, usually, there are frequent relapses and combination therapy may be of great help in this direction. Finally, HIV infection can present with dyskinesia/Parkinsonism, which may be very difficult to diagnose, and treat. The standard treatment of Parkinsonism in HIV patients is usually not beneficial. Besides other associations like diabetes and hyperuricaemia can occur which can be very difficult to treat.

## Consent

Written Informed consent was obtained from the patient's son, who was a graduate, for publication of this case report. A copy of the written consent is available for review by the Editor-in-Chief of the journal.

## Competing interests

The authors declare that they have no competing interests.

## Authors' contributions

KP recorded the patient data and performed clinical examination and management of the patient. VNR and NK helped in case managment. SB did the CD4 count of the patient. NV perfomed the haematogical and pathological test whereas CSL performed all the biochemical investigations. PKS and PD were the major contributors in writing the manuscript. All authors read and approved the final manuscript.
